# Cost-Effectiveness of the CNIC-Polypill Strategy Compared With Separate Monocomponents in Secondary Prevention of Cardiovascular and Cerebrovascular Disease in Portugal: The MERCURY Study

**DOI:** 10.36469/001c.39768

**Published:** 2022-11-22

**Authors:** Carlos Aguiar, Francisco Araujo, Gabriel Rubio-Mercade, David Carcedo, Silvia Paz, Jose María Castellano, Valentín Fuster

**Affiliations:** 1 Serviço de Cardiologia, Hospital de Santa Cruz, Carnaxide, Portugal; 2 Departamento de Medicina Interna, Hospital Lusíadas, Lisboa, Portugal; 3 Ferrer Internacional, Barcelona, Spain; 4 Hygeia Consulting, Madrid, Spain; 5 SmartWorking4U, Benicàssim, Spain; 6 Centro Nacional de Investigaciones Cardiovasculares, Instituto de Salud Carlos III, Madrid, Spain; 7 Centro Integral de Enfermedades Cardiovasculares, Hospital Universitario HM Monteprincipe, Grupo HM Hospitales, Madrid, Spain; 8 Icahn School of Medicine at Mount Sinai, New York, New York

**Keywords:** CNIC-Polypill, cost-effectiveness, cost-utility, secondary prevention, cardiovascular disease, cerebrovascular disease, cardiovascular risk factors

## Abstract

**Background:** Cardiovascular (CV) diseases remain a leading and costly cause of death globally. Patients with previous CV events are at high risk of recurrence. Secondary prevention therapies improve CV risk factor control and reduce disease costs.

**Objectives:** To assess the cost-effectiveness of a CV polypill strategy (CNIC-Polypill) compared with the loose combination of monocomponents to improve the control of CV risk factors in patients with previous coronary heart disease or stroke.

**Methods:** A Markov model cost-utility analysis was developed using 4 health states, SMART risk equation, and 3-month cycles for year 1 and annual cycles thereafter, over a lifetime horizon from the perspective of the National Health System in Portugal (base case). The NEPTUNO study, Portuguese registries, mortality tables, official reports, and the literature were consulted to define effectiveness, epidemiological costs, and utility data. Outcomes were costs (estimated in 2020 euros) per life-year (LY) and quality-adjusted LY (QALY) gained. A 4% discount rate was applied. Alternative scenarios and one-way and probabilistic sensitivity analyses tested the consistency and robustness of results.

**Results:** The CNIC-Polypill strategy in secondary prevention provides more LY and QALY, at a higher cost, than monocomponents. The incremental cost-utility ratio is €1557/QALY gained. Assuming a willingness-to-pay threshold of €30 000/QALY gained, there is a 79.7% and a 44.4% probability of the CNIC-Polypill being cost-effective and cost-saving, respectively, compared with the loose combination of monocomponents. Results remain consistent in the alternative scenarios and robust in the sensitivity analyses.

**Discussion:** The model reflects increments in the number of years patients would live and in quality of life with the CNIC-Polypill. The clinical effectiveness of the CNIC-Polypill strategy initially demonstrated in the NEPTUNO study has been recently corroborated in the SECURE trial. The incremental cost of the CNIC-Polypill strategy emerges slightly above the comparator, but willingness-to-pay estimates and sensitivity analyses indicate that the CNIC-Polypill strategy is consistently cost-effective compared with monocomponents and remains within acceptable affordability margins.

**Conclusion:** The CNIC-Polypill is a cost-effective secondary prevention strategy. In patients with histories of coronary heart disease or stroke, the CNIC-Polypill more effectively controls CV risk factors compared with monocomponents.

## BACKGROUND

Around 100 million people worldwide have established cardiovascular (CV) diseases,^1^ which remain the leading cause of death globally.^2^ Between 20% and 30% of these patients will suffer a recurrent myocardial infarction (MI), stroke, heart failure, or CV death within 5 years.^1^ Cardiovascular disease burden is increasing in terms of both deaths and disability-adjusted life-years, representing between 7.6% and 21.0% of national health expenditures around the world.^3^

Effective secondary prevention therapies can improve the quality of life of individuals with established CV diseases and reduce healthcare costs while increasing economic productivity.^3^ Effective secondary prevention of CV and cerebrovascular diseases, however, requires adequate assessment of risk and timely implementation of preventive interventions,^4^ including early intensification in high-risk persons.^5,6^ International guidelines indicate that the most effective cardioprotective drug therapy for secondary prevention includes low-dose aspirin, statins, and angiotensin-converting enzyme inhibitors (ACEi).^7,8^ Patients who routinely take this combination therapy have considerably lower risk of recurrent MI, strokes, transient ischemic attacks, and even death, compared with those receiving partial treatment with 1 or 2 of these drugs.^9^ However, the high complexity of preventive dosing regimens leads to suboptimal prescribing patterns and low adherence to cardioprotective medication.^10,11^ Simplifying treatment regimens with a fixed-dose combination polypill containing the recommended treatments in a single daily capsule has been proven to improve adherence and risk-factor control in secondary prevention patients.^11^

The polypill strategy developed by the Centro Nacional de Investigaciones Cardiovasculares (CNIC), henceforth the CNIC-Polypill, a fixed-dose combination pill containing 100 mg acetylsalicylic acid (ASA), 20/40 mg atorvastatin, and 2.5/5/10 mg ramipril, is indicated in the secondary prevention of CV events, as a replacement treatment in adult patients.^12^ The CNIC-Polypill has been developed using a patented structure that avoids physical and chemical interactions between the polypill components, maintaining all their biopharmaceutical and pharmacokinetic properties. It is the only CV polypill that has been granted regulatory approval in 28 countries worldwide.^13^ Based on its improved adherence, real-world studies have consistently shown its effectiveness, good tolerability. and affordability around the world.^14–16^

The CNIC-Polypill strategy improves CV risk factors in secondary prevention patients with previous history of CV diseases.^17–22^ The recent retrospective NEPTUNO study^23^ showed that, compared with monocomponents, the CNIC-Polypill strategy effectively reduces incidence of recurrent major adverse cardiovascular events (MACE), total cholesterol (TC), and systolic blood pressure (SBP) and increases high-density lipoprotein cholesterol (HDL-c). In the NEPTUNO study, the cohort treated with the CNIC-Polypill (cohort 1), compared with the identical monocomponents (cohort 2), equipotent medication (cohort 3) and usual care (cohort 4) cohorts, had a significant reduction in the incidence of recurrent MACE (22%; *P* = .017, 25%; *P =*.002, 27%; *P =*.001, higher in the monocomponents, equipotent and usual cohorts, respectively).^23^ Time to the event was also longer in the CNIC-Polypill cohort compared with the other 3 cohorts. The SECURE (Secondary Prevention of Cardiovascular Disease in the Elderly) study,^24^ a prospective, multicentric, randomized clinical trial, recently demonstrated that treatment with the CNIC-Polypill within 6 months after MI results in a significantly lower risk of recurrent MACE (CV death, acute MI, stroke, or urgent revascularization [hazard ratio: 0.76; (95% CI, 0.60-0.96), *P =*.02]) than usual care at a median of 3 years of follow-up. However, considering the effective control of CV risk factors, the cost-effectiveness of the CNIC-Polypill strategy has yet to be evaluated. Previous economic evaluations focused on the clinical benefits of better adhering to treatment with the CNIC-Polypill.^14,15^ Cost analyses derived from the NEPTUNO study have also shown that the use of the CNIC-Polypill results in significantly lower healthcare resource utilization and total and direct medical costs compared with the control cohorts but did not assess the incremental cost-effectiveness of using the CNIC-Polypill to control CV risk factors in usual clinical practice.^25^

The aim of this study is to assess the cost-effectiveness implications of more effectively controlling CV risk factors in the secondary prevention CV and cerebrovascular disease population with the use of the CNIC-Polypill compared with the concomitant use of the monocomponents in usual clinical practice.

## METHODS

### Economic Model Structure

A Markov model with 3-month-long cycles for the first year and annual cycles thereafter was developed and set for Portugal.^26–31^ A 3-month cycle was considered for the first year to increase spatial granularity^32^ and to reflect the most vulnerable 90-day windows for recurrent events after an acute coronary index event.^33^ Annual cycles were then considered according to the most frequently reported approach in pharmacoeconomic models for CV diseases.^34^

The Markov model assessed the cost utility of the CNIC-Polypill strategy compared with treatment with monocomponents administered concomitantly for the secondary prevention of CV events over a lifetime horizon. Cost-utility analysis is a form of cost-effectiveness analysis in which the outcomes are measured in terms of costs per quality-adjusted life-year (QALY) gained.^35^ The pharmacoeconomic model and analysis plan were discussed and agreed among the coauthors in advance.

The model assessed a hypothetical sample of 1000 individuals who closely replicated the baseline characteristics of the Portuguese population with previous coronary heart disease (CHD) or stroke in need of secondary CV event prevention (**Supplementary Table S1.1**).

**Figure 1.**
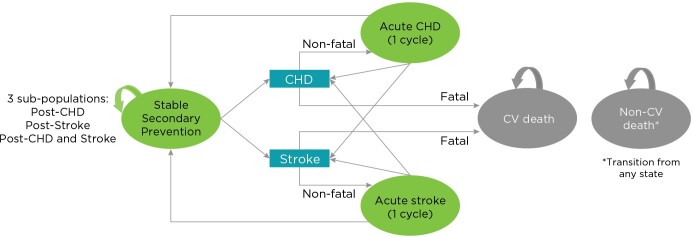
Model Structure Abbreviations: CHD, coronary heart disease; CV, cardiovascular. The possible health states included in the Markov model (stable secondary prevention; acute CHD; acute stroke; and death state [CV or non-CV]) apply to both treatment strategies: treatment with the cardiovascular polypill and with monocomponents.

The model consisted of 4 mutually exclusive health states: (1) “stable secondary prevention,” (2) “acute CHD,” (3) “acute stroke,” and (4) “death” state (CV or non-CV cause). All patients entered the model in the stable secondary prevention state, and in each cycle, patients could remain in this state or suffer a recurrent event (CHD or stroke) that would prompt their transition to either the acute CHD or the acute stroke state if the event was nonfatal, or to the death state if the event was fatal. Patients in the acute CHD or the acute stroke states remained there for a cycle before either returning to the stable secondary prevention state or suffering a subsequent CHD event or stroke, which, again, can be fatal or nonfatal and prompt their transition to the death state or would cause them to remain in either of the acute states for another cycle. Transition to the death state could occur from any state for causes other than CV events (**Figure 1**). Half-cycle correction was applied to the model to account for midcycle transitions.

Key CV risk equations described below determined the probability of transition between health states based on the modification of known CV risk factors (TC reduction, HDL-c increment, SBP reduction) stemming from treatment with either the CNIC-Polypill or monocomponents.

The analysis adopted the payer perspective of the Portuguese National Health Service (Serviço Nacional de Saúde). A 4% discount rate was applied to the health outcomes and cost results on a yearly basis after the first year.^36^

Outcomes were expressed in terms of incremental cost-effectiveness ratio (ICER) per life-year (LY) and QALY gained for the CNIC-Polypill vs care with monocomponents. Additionally, cost outcomes were expressed as total costs per patient, and health outcomes as LYs and QALYs gained, number of subsequent/recurrent events, and CV deaths prevented (**Table 1**).

**Table 1.** Characteristics of the Cost-effectiveness Model

Population	Secondary prevention patients who have suffered a CHD event or a stroke (cohort weighted 57.9% post-CHD and 42.1% post-stroke)
Intervention	Polypill strategy (ASA, atorvastatin, ramipril)
Comparators	Same monocomponents administered concomitantly^a^
Model structure	Markov model with 4 health states Stable Acute CHD Acute stroke Death state (CV death, non-CV death) Annual cycles (except for the first year with 3-month cycle length)
Model setting	Payer perspective of the Portuguese National Healthcare Service Lifetime horizon Discount rate (costs and health outcomes): 4%
CV risk equations	SMART risk equations for recurrent CHD and stroke risk model (Dorreijstein et al^37^) for the base case Framingham risk equations for the sensitivity analysis D’Agostino et al^38^ for subsequent CHD risk model D’Agostino et al^39^ for primary stroke risk model
Outcomes	Health outcomes: LY gained QALY gained No. of subsequent CV events, recurrent strokes, and CV deaths prevented by using the polypill strategy vs monocomponents Cost outcomes: Total costs Incremental results ICER with polypill strategy vs monocomponents ICUR with polypill strategy vs monocomponents

Abbreviations: ASA, acetylsalicylic acid; CHD, coronary heart disease; CV, cardiovascular; ICER, incremental cost-effectiveness ratio; ICUR, incremental cost-utility ratio; LY, life-year; QALY, quality-adjusted life-year; SMART, Secondary Manifestations of Arterial Disease. ^a^Cohort 2 of the NEPTUNO study in which patients received exactly the same monocomponents as the CNIC-Polypill.

### Transition Probabilities

Transition probabilities through the different health states were derived from event rates determined by a set of CV risk equations incorporated in the model.^26–31^ Diverse CV risk algorithms will estimate CV risk differently, resulting in significant heterogeneity in the clinical management of these groups of patients and in the analysis of the cost-effectiveness of the strategies used to treat them.^40,41^ Bearing this in mind, the model incorporated 2 different CV risk equations, the Secondary Manifestations of Arterial Disease (SMART)^37^ as the base case and the Framingham Study^38,39^ as a sensitivity analysis to assess the robustness of the cost-effectiveness results **(Supplementary File 2)**. The SMART risk equation was selected for the base case in this economic model because it was developed to predict recurrent CV and cerebrovascular events in a population of patients with previous CV diseases in Europe (the Netherlands).^37^ The SMART risk equation has been externally validated in multiple countries^5^ and therefore has high generalizability. The SMART risk equation is based on age, gender, and clinical parameters in a Cox proportional hazards model to calculate the occurrence of major CV events (MI, stroke, or CV death):

**Figure:**
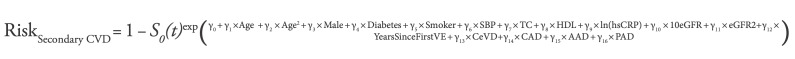


where SBP is systolic blood pressure, TC is total cholesterol, HDL is high-density lipoprotein, hsCRP is high-sensitivity C-reactive protein, eGFR is estimated glomerular filtration rate, VE is vascular event, CeVD is cerebrovascular disease, CAD is coronary artery disease, AAD is antiarrhythmic drugs, and PAD is peripheral arterial disease. Since the *S_0_* estimate corresponds to a period of 10 years, risk estimates obtained with the SMART risk equation were adjusted to the cycle-length assuming a constant rate over the 10-year period (**Supplementary Table S1.2**). Non-CV-related mortality was determined by general population mortality tables.^42^

### Inputed Data

A series of systematic literature reviews were completed to identify clinical effectiveness, epidemiological, cost, and utility data. Systematic searches were conducted in PubMed and Scopus for relevant studies published in English or in Portuguese up to November 30, 2020. The review of the literature was conducted in accordance with the Preferred Reporting Items for Systematic Review and Meta-Analysis (PRISMA) guidelines.^43^ The literature search initially identified 3063 publications (**Supplementary File 3**). Of these, 55 full-text versions of publications (15 effectiveness, 23 epidemiology, 9 cost, 8 health utility) were retrieved and appraised by reviewers, who short-listed a total of 28 studies that presented suitable data to populate the pharmacoeconomic model. Researchers and clinical experts checked the short-listed studies for relevance and selected the ones which would ultimately be used to inform the model. Additionally, clinical experts identified renowned international publications that provided data applicable to the Portuguese setting (**Table 2**).

**Table 2.** Summary of Base Case Inputs for Portugal

	**Base Case**	**Source**
Time horizon	Lifetime	—
Discount rate	4%	Perelman et al^36^
Payer perspective	NHS of Portugal	—
Willingness-to-pay threshold	€30 000	Perelman et al,^36^ NICE^44^
Population data^a^		
Starting age, y	69.37	Timóteo et al,^45^ Santos et al^46^
Male, %	61.8	Timóteo et al,^45^ Santos et al^46^
Smokers, %	17.4	Timóteo et al,^45^ Santos et al^46^
Diabetes, %	27.8	Timóteo et al,^45^ Santos et al^46^
Atrial fibrillation, %	13.2	Timóteo et al,^45^ Santos et al^46^
Left ventricular hypertrophy, %	6.2	Assumed equal to HF % from Castellano et al,^17^ Santos et al^46^
Systolic blood pressure, mm Hg	145.1	Ferreira,^47^ Heuschmann et al^48^
Total cholesterol, mg/dL	190.8	Ferreira,^47^ Heuschmann et al^48^
HDL-cholesterol, mg/dL	45.2	Ferreira,^47^ Heuschmann et al^48^
hs-CRP, mg/dL	2.2	Dorresteijn et al^37^
eGFR, ml/min/1.73m^2^	74.3	Timóteo et al,^49^ Dorresteijn et al^37^
History of abdominal aortic aneurysm, %	2.1	Castro-Ferreira et al^50^
History of peripheral arterial disease, %	3.2	Timóteo et al^51^ Santos et al^46^
Polypill effectiveness		
SBP reduction, mm Hg	1.80	NEPTUNO, cohort 2^52^
TC reduction, mg/dL	5.28	NEPTUNO, cohort 2^52^
HDL-c increment, mg/dL	4.01	NEPTUNO, cohort 2^52^
Health utilities		
Chronic CHD	0.84	Ara et a^53^
Chronic stroke	0.69	Ara et a^53^
Acute CHD	0.76	Ara et a^53^
Acute stroke	0.63	Ara et al^53^
Death	0.00	Assumption
Drug costs (€)		
Annual reimbursement cost—polypill^b^	84.62	Calculated based on Infarmed drug database
Annual reimbursement cost—monotherapies	63.44	Calculated based on Infarmed drug database
CV event management costs (€)		
Cost of acute CV event (nonfatal)	4560.1	Costa et al^54^
Cost of acute CV event (fatal)	3153.5	Silva Miguel and Ferreira^55^
Cost of acute stroke (nonfatal)	8653.3	Costa et al^54^
Cost of acute stroke (fatal)	6381.2	Costa et al^54^
Annual cost post-CV event	643.3	Costa et al^54^
Annual cost post-stroke	534.8	Costa et al^54^
SMART 10-y CV risk		
*S_0_*(*t*=10), adjusted to the cycle length assuming a constant rate over the 1 y and lifetime periods	0.8107	Dorresteijn et al^37^

Abbreviations: CHD, coronary heart disease; eGFR, estimated glomerular filtration rate; HDL-c, high density lipoprotein cholesterol; HF, heart failure; hs-CRP, high sensitivity C-reactive protein; NICE, National Institute for Health Care and Excellence; SBP, systolic blood pressure; SMART, Secondary Manifestations of Arterial Disease; TC, total cholesterol. ^a^Population data weighted 57.9% and 42.1% from individual post-CHD and post-stroke cohorts, respectively. ^b^Since all healthcare resources considered must be aligned with the National Health Service perspective, only reimbursed medication costs were included and therefore patient copayments were excluded. Assumptions were made when data was unavailable or inconsistent (**Supplementary Table S1.3**). Assumptions were validated by the clinical experts.

### CNIC-Polypill Effectiveness

Effectiveness data was retrieved from NEPTUNO,^23^ a retrospective observational study that assessed the clinical effectiveness (control of CV risk factors and incidence of recurrent MACE), resource use, and healthcare costs of the CNIC-Polypill strategy vs a loose combination of identical monocomponents or therapeutic equivalents in routine clinical practice for secondary prevention in Spain. According to the opinion of experts, the NEPTUNO study was chosen to populate the economic model due to the similar CV risk that exists between the Portuguese and the Spanish populations.^2^

In NEPTUNO,^23^ cohort 1 received the CNIC-Polypill strategy, and cohorts 2, 3, and 4 received different loose combinations of monocomponents. In cohort 2, all patients received the same 3 monocomponents and strengths as the CNIC-Polypill; in cohort 3, all patients received therapeutic alternatives of equivalent potency to the monocomponents of the CNIC-Polypill^56,57^; in cohort 4, patients could receive 1, 2, or 3 monocomponents or therapeutic alternatives that may or may not be of equivalent potency to the CNIC-Polypill, representing a usage of 86.1%, 70.7%, and 87.7% of antiplatelets, statins, and antihypertensive medication, respectively (**Supplementary Table S1.4**). A propensity-score matching was performed to ensure comparability among the different cohorts.^23^

The NEPTUNO study showed that the control of CV risk factors significantly improved in the CNIC-Polypill cohort compared with the other 3 cohorts during the 2-year follow-up. There were significant reductions in TC (174.6 mg/dL vs 187.2 mg/dL, 198.0 mg/dL, and 198.1 mg/dL, respectively, for cohorts 2, 3, and 4; *P* < .001) and in SBP (126.5 mm Hg vs 128.3 mm Hg, 129.1 mm Hg, and 123.1 mm Hg, respectively, for cohorts 2, 3, and 4; *P* < .001) and an increase in HDL-c (55.4 mg/dL vs 53.0 mg/dL, 53.0 mg/dL, and 51.8 mg/dL, respectively, for cohorts 2, 3, and 4; *P* < .001). The comparative clinical effectiveness results of the CNIC-Polypill strategy vs each of the cohorts are summarized in **Supplementary Table S1.5**.

For the base case of this analysis, cohort 2 (identical monocomponents) was selected as the comparator while cohorts 3 (equipotent therapeutic alternatives) and 4 (other treatments) were used in the scenario analysis to test the consistency of the results. The selection of NEPTUNO cohort 2 as the comparator in the base case scenario is a conservative approach chosen to more coherently compare the CNIC-Polypill strategy against the same monocomponents given separately.

### Costs

Only direct costs were considered, including acquisition costs of medications, costs of managing acute CV disease and cerebrovascular events, and costs of follow-up after hospital discharge. All costs were reported in 2020 euros (€).

Annual medication costs were calculated using the pharmacy selling price with value-added tax published in the Infarmed drug database.^58^ Since all healthcare resources to be considered must be aligned with the National Health Service perspective, only reimbursed medication costs were included; therefore, patient copayments were excluded.^36^ There are several presentations of the CNIC-Polypill (fixed-dose ASA with variable doses of atorvastatin and ramipril) and, for the base case of this analysis, the costs of all available presentations were weighted according to their use in NEPTUNO cohort 1. For the monocomponents, Portuguese reference prices were used and the costs of all the presentations in each class of drugs (antiplatelets, statins, and antihypertensive medication) were weighted according to the patients in the different NEPTUNO cohorts (**Supplementary Table S1.6**).

The literature search strategy provided Portuguese event management direct costs (acute and follow-up). Acute costs include the management of the acute episode and during the first year (**Supplementary Table S1.6**).^54,55^

### Health Utilities

Health utility values were considered for 4 health states: (1) chronic CHD or chronic stroke for those patients stable on secondary prevention, (2) acute CHD or (3) acute stroke for those who suffer a recurrent event, and (4) death (**Supplementary Table S1.7**). When patients suffered a nonfatal event, utility for an acute event was applied for a duration of one Markov cycle (for 3 months during the first year and for a duration of 1 year thereafter). For the second and subsequent years, utility weights relating to chronic states were used, with the lower of the 2 utilities being applied for patients who had suffered both CHD and stroke events.

Health utility values from Ara et al^53^ were selected for the base case because they are frequently used in economic evaluations of secondary prevention of CV diseases.^59,60^ They were obtained from the 3-level EuroQol self-report questionnaire (EQ–5D-3L) collected on individuals in the United Kingdom. The literature search strategy did not identify any studies that considered utility estimates from the Portuguese population (**Supplementary Table S1.7**).

### Sensitivity Analyses and Alternative Scenarios

Both deterministic one-way sensitivity analysis (OWSA) and probabilistic sensitivity analysis (PSA) were performed to evaluate the impact of parameter uncertainty on the robustness of the results. One-way sensitivity analysis was conducted for all major model variables to explore the effects on the ICER results. All parameters included in the OWSA were modified ±20% (assumed standard error in the absence of variability data) from the base case value. Results were presented as tornado diagrams. In the PSA, a Monte Carlo simulation was conducted using 1000 iterations based on model inputs randomly drawn from distributions around the mean. Normal distribution for population characteristics data, beta distribution for population characteristics expressed as rates or percentages, clinical effectiveness and utility data, and a gamma distribution for costs were applied. A normal distribution was also used in the simulation for all CV risk equations except for the relative risk of recurrent stroke, where a lognormal distribution was employed. The PSA results were depicted in cost-effectiveness acceptability curves and scatterplot planes.

Additionally, the following alternative scenario analyses were performed:

- Varying the time horizon to 2, 5, 10, and 20 years, instead of a lifetime horizon (32 years)
- Using Framingham risk equations instead of the SMART risk equation
- Using the monocomponents distribution and effectiveness information from the other 2 NEPTUNO cohorts: cohorts 3 and 4
- Running 5000 and 10 000 iterations in the PSA

### Ethical Considerations

This was a pharmacoeconomic, noninterventional study, based on the collection of data from the literature and from public health reports and registries without involvement of human subjects. Ethical approval was not required to conduct this study.

### Reporting Considerations

The Consolidated Health Economic Evaluation Reporting Standards 2022 (CHEERS 2022) checklist was used to report on the results of this cost-effectiveness analysis (**Supplementary Table S1.8**).^61^

## RESULTS

### Base Case

Base case results are displayed in **Table 3**. The use of the CNIC-Polypill strategy over the lifetime of a cohort of 1000 patients who have suffered either a CHD event or a stroke prevents 17 subsequent CHD events, 15 recurrent strokes, and 6 CV deaths vs the loose combination of monocomponents. This translates into more LY and QALY gained (39 additional LY and 33 extra QALY), although at a higher cost (€51 802), which is mainly due to the increases in pharmacological expenditure during follow-up. Nevertheless, the cost-effectiveness and cost-utility ratios are well below the cost-effectiveness threshold of €10 000/QALY to €100 000/QALY recommended by methodological guidelines for economic evaluation studies of health technologies in Portugal^36^ and the €30 000/QALY threshold usually considered in the rest of Europe^44^: €1327/LY gained and €1557/QALY gained.

**Table 3.** Cost-effectiveness Results for the Weighted Cohort in the Base Case Analysis and Alternative Scenarios

	**Base Case**			**Alternative Scenarios (Polypill vs Monocomponents)**						
	**Polypill**	**Monocomponents**	**Polypill vs Monocomponents**	**2-y Horizon**	**5-y Horizon**	**10-y Horizon**	**20-y Horizon**	**Framingham Risk Equation**	**NEPTUNO Cohort 3**	**NEPTUNO Cohort 4**
Total costs (€)	10 940 008	10 888 206	+51 802	+23 888	+50 289	+72 087	+59 590	+72 705	-22 958	-63 287
Drug costs (€)	875 140	653 763	+221 376	+39 910	+91 160	+155 546	+215 715	+219 292	+252 198	+236 203
CV event management cost (acute event)(€)	3 820 390	4 010 119	-189 729	-16 282	-42 415	-89 216	-173 343	-175 818	-307 749	-334 938
Subsequent nonfatal CHD (€)	1 124 769	1 180 641	-55 872	-4764	-12 415	-26 142	50 960	-114 691	-90 626	-98 632
Subsequent fatal CHD (€)	212 844	223 404	-10 560	-930	-2420	-5067	-9715	-21 632	-17 129	-18 643
Recurrent nonfatal stroke (€)	2 156 242	2 263 323	-107 081	-9 195	-23 953	-50 377	-97 850	-34 301	-173 691	-189 036
Recurrent fatal stroke (€)	32 535	342 751	-16 216	-1392	-3627	-7629	-14 511	-5194	-26 303	-28 627
CV event management cost (follow-up)(€)^a^	6 244 478	6 224 323	+20 154	+260	+1 545	+5 757	+17 218	+29 231	+32 593	+35 448
No. of subsequent CV events	306	323	-17	-1	-3	-7	-14	-31	-27	-29
No. of recurrent strokes	300	315	-15	-1	-3	-7	-14	-5	-24	-26
No. of CV deaths	119	125	-6	-0.5	-1	-3	-5	-8	-10	-10
LY	9760.83	9721.80	39.04	0.89	4.28	13.80	35.20	59.28	63.20	68.75
QALY	7371.46	7338.20	33.26	0.83	3.89	12.25	30.26	46.29	53.89	58.63
ICER (€ per LY gained)			1327	26 775	11,738	5225	1693	1226	Polypill dominant	Polypill dominant
ICUR (€ per QALY gained)			1557	28 885	12 934	5884	1969	1571	Polypill dominant	Polypill dominant
Probability cost-effective (PSA)^a^			79.7%	54.6%	65.4%	71.2%	82%	97%	87.8%	88.8%

Abbreviations: CHD, coronary heart disease; CV, cardiovascular; ICER, incremental cost-effectiveness ratio; ICUR, incremental cost-utility ratio; LY, life-year; PSA, probabilistic sensitivity analysis; QALY, quality-adjusted life-year. ^a^Applied in each year of the simulation after the first acute event. ^b^Assuming a willingness-to-pay threshold of €30 000/QALY gained.

### Sensitivity Analysis

**Figure 2.**
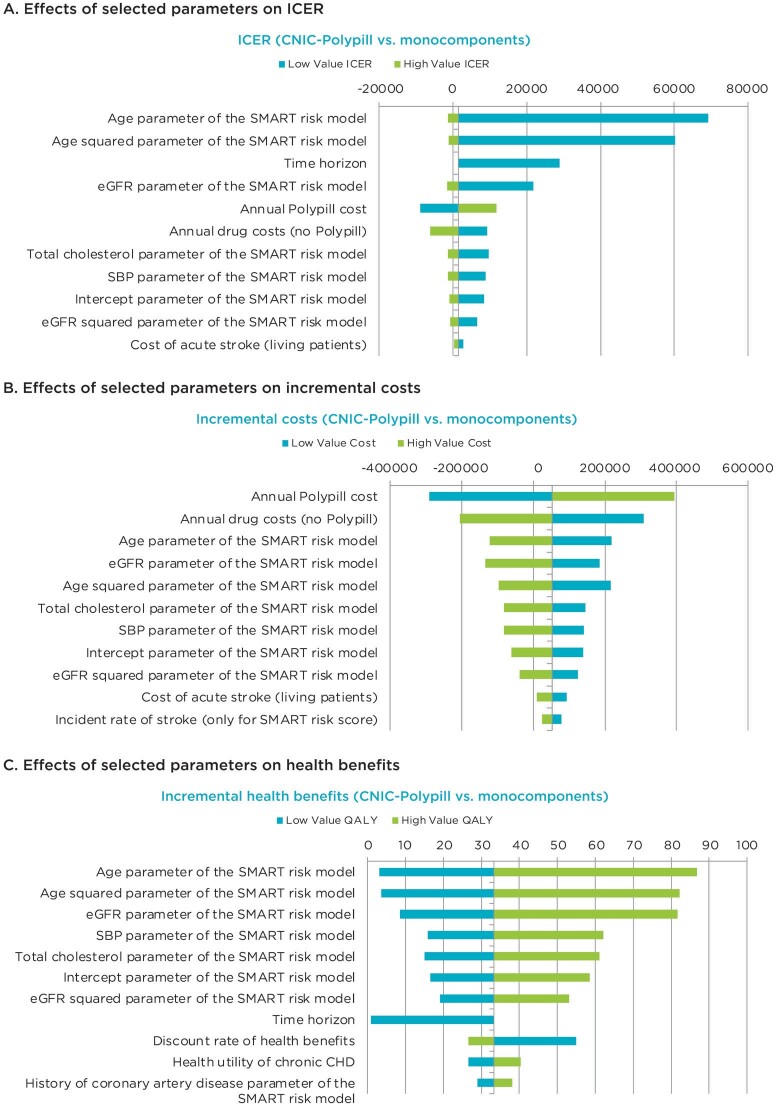
One-way Sensitivity Analyses Results Abbreviations: CHD, coronary heart disease; eGFR, estimated glomerular filtration rate; HDL, high-density lipoprotein; ICER, incremental cost-effectiveness ratio; SBP, systolic blood pressure; SMART, Secondary Manifestations of Arterial Disease; TC, total cholesterol. Impact of low and high values of each variable on the ICER (**A**), incremental costs (**B**), and incremental health benefit results (**C**) of the base case. The ICER and incremental health benefit results are most sensitive to the age parameters, whereas the incremental cost results are most sensitive to the annual cost of the CNIC-Polypill.

**One-way sensitivity analysis:** One-way deterministic sensitivity analyses, shown in the tornado diagrams in **Figure 2**, demonstrate that the ICER (**Figure 2A**) and incremental health benefits (**Figure 2C**) results are most sensitive to the age parameters, whereas the incremental cost results (**Figure 2B**) are most sensitive to the annual cost of the polypill.

**Figure 3.**
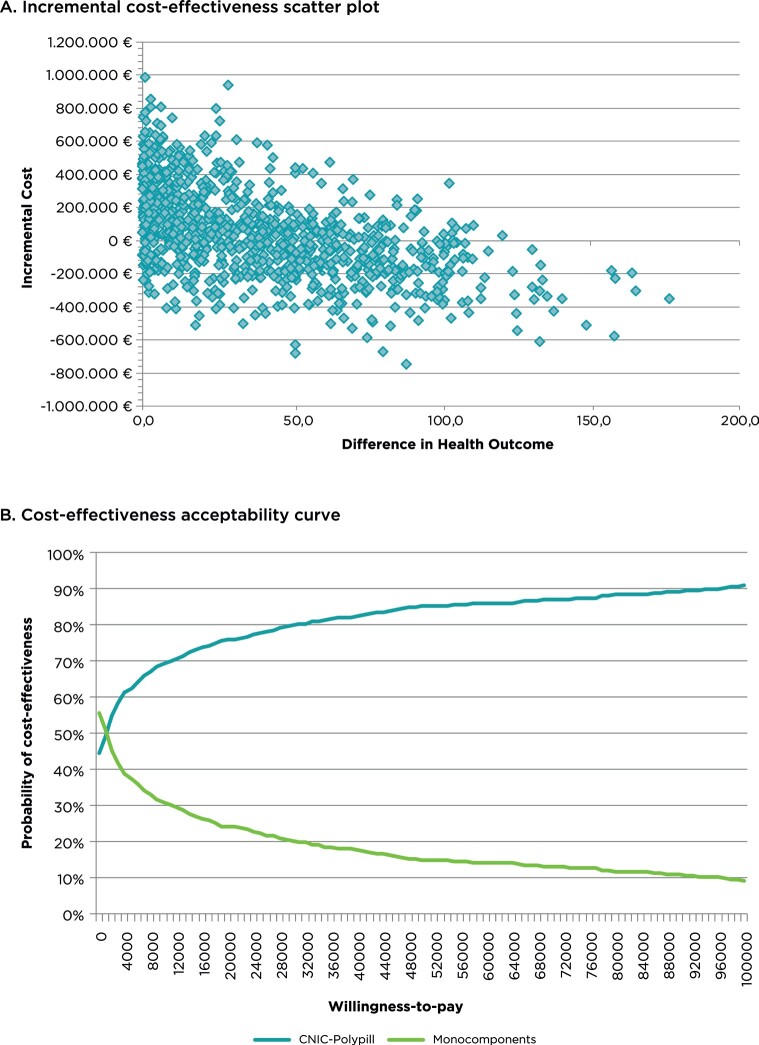
Probabilistic Sensitivity Analysis Results Abbreviation: PSA, probabilistic sensitivity analysis. (**A**) The incremental cost of the CNIC-Polypill monocomponents is represented on the y-axis and the incremental health outcomes as QALYs on the x-axis. The average increase in QALY obtained from the 1000 simulations is 38.88 (0.41-113.31), while the average cost increase is €52 390 (€-424 619 to €590 103). The average ICUR is €1347/QALY, which confirms the robustness of the results of the base case of the analysis. (**B**) The y-axis represents the probability that the simulations performed exceed the willingness-to-pay threshold, which is represented in the x-axis, expressed in euros (€) per QALY gained. As the willingness-to-pay increases (along the x-axis), the probability that the CNIC-Polypill strategy is the most cost-effective option is also greater. The probability of the CNIC-Polypill being the most cost-effective option begins at low pay values (from €2000/QALY gained). Furthermore, assuming a willingness-to-pay threshold of €30 000/QALY gained, the PSA indicates that there is a 79.7% probability that the CNIC-Polypill is a cost-effective treatment with a loose combination of monocomponents.

**Probabilistic sensitivity analysis**: Probability sensitivity analysis showed that the average incremental cost-utility ratio (ICUR) obtained from the 1000 simulations is €1347/QALY gained, confirming the robustness of the base case results (**Figure 3A**). Furthermore, assuming a willingness-to-pay threshold of €30 000/QALY gained, the PSA indicates that there is a 79.7% probability that the CNIC-Polypill strategy is cost-effective and a 44.4% probability that it is cost-saving compared with the loose combination of monocomponents (**Figure 3B**).

### Scenario Analyses

Scenario analysis indicated the following:

- Varying the time horizon to 2, 5, 10, and 20 years shows progressively increasing ICURs with shorter time horizons, with €1969/QALY gained at 20 years, €5884/QALY gained at 10 years, €12 934/QALY gained at 5 years, and €28 885/QALY at the 2-year time horizon. Likewise, the probability of the CNIC-Polypill strategy being cost-effective, assuming a willingness-to-pay threshold of €30 000/QALY gained, increases with longer time horizons, starting at 54.6% when the time horizon selected is 2 years and reaching 82% at a 20-year time horizon.
- Using Framingham instead of the SMART risk equation results in the CNIC-Polypill strategy offering additional LY and QALY gained compared with the base case (59 LY and 46 QALY), although at a higher incremental cost (€72 705). This scenario results in very similar ICERs and ICURs to those seen in the base case, with €1226/LY and €1571/QALY gained. Assuming a willingness-to-pay threshold of €30 000/QALY gained, with the Framingham risk equation there is a 97% probability of the CNIC-Polypill strategy being cost-effective and a 42.2% probability of it being cost-saving compared with the loose combination of monocomponents.
- Using the different monocomponent distributions and effectiveness results from NEPTUNO cohort 3, in which all patients received monocomponents of equivalent potency to the CNIC-Polypill, and cohort 4, the closest to real-life clinical practice, in which patients could receive 1, 2, or 3 monocomponents that could be of the same or different potency to those of the CNIC-Polypill, results in the CNIC-Polypill strategy being dominant over the respective monocomponent combinations, which means it is more effective at providing health benefits at a lower cost.
- Running 5000 and 10 000 iterations in the PSA show that cost-utility results remain consistent. At 5000 iterations there is 81.9% probability for the CNIC-Polypill to be cost-effective and 45.7% to be cost-saving. At 10 000 iterations, there is an 82.7% probability for the CNIC-Polypill to be cost-effective and 45.7% to be cost-saving.

The results of the scenario analyses are included in detail in **Table 3.**

## DISCUSSION

Our economic model evaluates the potential public health benefits and cost-effectiveness of the CNIC-Polypill (100 mg ASA, 20/40 mg atorvastatin, and 2.5/5/10 mg ramipril) strategy vs different combinations of monocomponents administered concomitantly for the treatment of secondary CV prevention patients from the perspective of the Portuguese healthcare system. The results show that treatment with the CNIC-Polypill strategy, based on its greater clinical effectiveness over monocomponents at improving blood pressure and lipid profile and reducing the CV risk, could avoid around 32 recurrent nonfatal events and 6 CV deaths per 1000 post-CHD or stroke patients over a lifetime horizon compared with a loose combination of its monocomponents. As a consequence of these gains in prevention, the model reflects increments in the number of years patients would live and in quality of life. The clinical effectiveness of the CNIC-Polypill strategy in secondary prevention initially demonstrated in the NEPTUNO study^23^ has been recently corroborated in the SECURE trial.^24^ First, the NEPTUNO study^23^ showed that after 2 years of follow-up the cumulative incidence of recurrent MACE was lower and the incremental proportion of patients who achieved adequate control of BP and low-density lipoprotein cholesterol from baseline was higher in the CNIC-Polypill cohort compared with control cohorts.

Recently, the SECURE trial^24^ showed that after a median of 3 years of treatment, CV death occurred less frequently among patients in the CNIC-Polypill group compared with those in the usual care group (HR: 0.67; 95% CI, 0.47-0.97). These findings support the validity of our effectiveness results in the model.

The incremental cost of the CNIC-Polypill strategy emerges slightly above the comparator, but willingness-to-pay estimates and sensitivity analyses indicate that the CNIC-Polypill strategy is consistently cost-effective compared with monocomponents and remains within acceptable affordability margins whenever variable and widely accepted thresholds for chronic conditions are considered.^36,44^ Subsequent cost-effectiveness analyses incorporating the SECURE trial results will inform about the incremental cost-effectiveness of avoiding CV death and improving survival among secondary prevention patients.

Our results are in line with other economic analyses of the CNIC-Polypill. Two previous studies, one in the United Kingdom^59^ and one in Spain,^60^ evaluated the cost-effectiveness of the same 3-component polypill (ASA, a moderate-potency statin, and an ACEi) vs the individual monocomponents prescribed simultaneously. Unlike the present study, in which effectiveness is based on the improvement of blood pressure and lipid profile, in these 2 previous studies, the health benefits were based on the improved adherence achieved with a 3-in-1 CNIC-Polypill compared with the adherence to the individual monocomponents. They both reported cost-effectiveness of the CNIC-Polypill at 10 years in exclusively post-MI patients. In the UK, the polypill strategy prevented 15% of fatal and nonfatal recurrent CV events per 1000 patients at an ICUR of £8200 per QALY gained.^59^ In Spain, use of the CNIC-Polypill strategy was dominant over its monocomponents prescribed concomitantly, avoiding 46 nonfatal and 11 fatal CV events per 1000 patients treated.^60^

The affordability of a 4-component polypill (ASA, a moderate- or low-potency statin, an ACEi, and a β-blocker) has also been demonstrated in 5 low-income and middle-income countries. A Markov model based on a 32% increase in adherence with the polypill strategy predicted that 40 to 54 major adverse CV events per 1000 patients treated over 5 years would be prevented in China, India, Mexico, Nigeria, and South Africa. The increment in costs was offset by the health benefits obtained from preventing recurrent events with the use of the polypill.^16^ Further studies in the United States^62,63^ and in India^64^ also proved the cost-effectiveness of a 4-component polypill (ASA, a moderate- or low-potency statin, an ACEi, and a β-blocker) compared with the monocomponents administered concomitantly after improving adherence to therapy. In contrast to these economic models, our study relies on the higher clinical effectiveness of the CNIC-Polypill strategy to decrease CV risk in a population in need of preventing subsequent events.

The strengths of our analysis include the use of NEPTUNO as the clinical study from which the effectiveness data have been retrieved.^25,52^ Real-world observational studies such as NEPTUNO, while being representative of real clinical practice, usually lack the strict parameter control of a clinical trial and are therefore unable to accurately provide a comparison between 2 strategies. However, the strength of NEPTUNO lies in its ability to provide this comparison through the patients in cohort 2, of which 100% received the identical monocomponents as the polypill. Furthermore, the other 2 cohorts in this study were able to provide a real-life representation of monocomponent use in clinical practice and allowed for a sensitivity scenario analysis in which the effect of the CNIC-Polypill was compared with other realistic treatment scenarios to assess the robustness of the base case results. In both alternative scenarios, the CNIC-Polypill emerges as the dominant strategy over the respective combinations of monocomponents.

Our pharmacoeconomic model is unique in analyzing the cost-utility of the CNIC-Polypill strategy in a stroke population as, to our knowledge, only 1 previous economic study^16^ targeted this population, with all other pharmacoeconomic assessments focusing on the effects on ischemic heart disease or post-MI populations.^59,60,62–64^ An additional distinctive feature of our model is the application of 2 different CV risk equations to determine transition probabilities between health states. Cardiovascular risk estimates differ significantly depending on the risk equation used and the population studied, determining varied pharmacoeconomic results.^41^ Thus, as expected, the cost-utility results of our model differ slightly when the SMART or the Framingham risk equations are employed. However, the ICUR differences after applying either risk equation are minimal, confirming the cost-effectiveness of the CNIC-Polypill strategy over monocomponents for the secondary prevention of CV and cerebrovascular disease and dispelling any uncertainty on the consistency of findings.

Further alternative scenarios show the model to be most sensitive to the time horizon being shortened. For time horizons shorter than 10 years, ICURs are considerably increased to over €10 000/QALY gained. This is thought to be due to shorter time horizons not capturing the full benefit of reducing the risk of CV events in the long term. Variations in monocomponents distribution within the NEPTUNO study result in the CNIC-Polypill strategy being dominant over monocomponents both when all patients receive monocomponents of equivalent potency to the polypill and when patients receive 1, 2, or 3 monocomponents that can be of the same or different potency to that of the polypill constituents. This could be due to a poorer CV risk factor control that may stem from using different monocomponents and fewer concomitant drugs. Despite variations, and barring the results obtained for short time horizons, all of the ICUR results in the scenarios analyzed are well below the cost-effectiveness threshold of €10 000 to €100 000/QALY usually considered in Portugal^36^ and the €30 000/QALY threshold usually considered in the rest of Europe,^44^ certifying the robustness of the base case results.

Like other studies based on modeling, this pharmacoeconomic assessment has a series of limitations that warrant mention. There are inherent limitations to all Markov models that stem from their structural rigidity, hindering a complete representation of usual clinical practice. The model’s long-term CV event prediction over a lifetime horizon is always subject to uncertainties. The SMART risk equation presents an inherent limitation in that it predicts a composite of recurrent vascular events (MI, stroke, and CV death). Country-specific MI and stroke incidence rates were employed to differentiate between recurrent CHD and stroke events. Neither the SMART nor the Framingham risk equations differentiate between fatal and nonfatal CV events, and therefore fatality event rates were obtained from the literature.^65,66^ Neither equation considers the cumulative risk of patients that have already suffered recurrent events, nor is this contemplated in the utility values, for which an incremental disutility weight would be required. Although the risk from cumulative events could have been built by including more health states and tunnel states, it is beyond the scope of this pharmacoeconomic analysis, which is devoted to showing the incremental costs and effectiveness effects that may occur as a consequence of more effectively controlling CV risk factors. A subsequent pharmacoeconomic assessment based on the recently published SECURE trial results would render complementary prospective cost-effectiveness evidence from reducing MACE events and CV death with the use of the CNIC-Polypill.

Another limitation results from the absence of a specific Framingham equation for predicting recurrent stroke and, therefore, from using a relative risk of recurrent vs primary stroke calculated from the literature.^66^ Estimating non-CV-related mortality from general population mortality tables is a further limitation. It may underestimate non-CV-attributable mortality due to patients with CV disease presenting a higher risk of death due to comorbidities compared with the general population.

An additional limitation of our model is that it does not consider the reduction in copayment costs for low-income retired patients. However, this limitation is negligible since it is expected that this change in costs would affect all cohorts in a similar way. Likewise, the price of the CNIC-Polypill may vary widely across countries, and adaptations of the model to each specific context may be required. The perspective is that of the National Health Service in Portugal instead of the wider societal perspective recommended in health economic guidelines.^67^ The use of healthcare resources and costs were estimated to represent this perspective of the payer.^61^

Besides limitations, the results of our pharmacoeconomic study show that the use of the CNIC-Polypill strategy in secondary prevention patients with a history of CHD or stroke is cost-effective compared with the use of a loose combination of identical monocomponents. The CNIC-Polypill strategy reduces both CV and cerebrovascular recurrent events at a higher cost but within widely accepted affordability thresholds. Furthermore, secondary prevention patients treated with the CNIC-Polypill have shown in previous studies a significantly higher degree of satisfaction and substantially increased medication adherence compared with patients that were treated with the monocomponents separately,^68^ which would further improve the potential health benefits already discussed.

This cost-utility study demonstrates the additional pharmacoeconomic gains to be obtained from further reducing CV risk factors with the use of the CNIC-Polypill strategy as baseline therapy in the secondary prevention population in real clinical practice.

### Author Contributions

G.R.M., S.P., F.A., and D.C. designed the pharmacoeconomic study. G.R.M., F.A., and S.P. contributed to the data acquisition. C.A., F.A., G.R.M., D.C., S.P., J.M.C., and V.F. all contributed to the interpretation of data, manuscript revision, and approval of the final manuscript.

### Disclosures

The authors declare the following potential conflicts of interest with respect to the research, authorship, and/or publication of this article: F.A. and G.R.M. were full-time employees of Ferrer, the manufacturer of the CNIC-Polypill, at the time of study development. C.A., J.M.C., and V.F. have received consulting fees from Ferrer. D.C. and S.P. received professional fees from Ferrer to develop the pharmacoeconomic model (D.C.) and to review the literature and provide writing and editorial assistance (S.P.).

### Presentations

This work was presented at the European Society of Cardiology (ESC) 2021 Virtual Congress (*Eur J Prev Cardiol.* 2021; 28(suppl 1):i434; the ESC Preventive Cardiology 2021 Virtual Congress (*Eur Heart J.* 2021; 42(suppl 1):ehab724.2552, *Eur J Prev Cardiol*. 2021;28(suppl 1):zwab061.446; the Congresso Português de Cardiologia 2021 (*Rev Port Cardiol*. 2021;40(Espec Congr):88-216 [oral presentation], *Rev Port Cardiol*. 2021;40(Espec Congr):88-216 [virtual poster] ; and the 8th European Stroke Organisation Conference (ESOC) 2021 Virtual Conference (O0174/#1090 Scientific Communication 023. *Eur Stroke J.* 2021;6(suppl 1):3-513. d.

## Figures and Tables

**Figure:** Online Supplementary Material
